# CFP1 coordinates histone H3 lysine-4 trimethylation and meiotic cell cycle progression in mouse oocytes

**DOI:** 10.1038/s41467-018-05930-x

**Published:** 2018-08-28

**Authors:** Qian-Qian Sha, Xing-Xing Dai, Jun-Chao Jiang, Chao Yu, Yu Jiang, Junping Liu, Xiang-Hong Ou, Song-Ying Zhang, Heng-Yu Fan

**Affiliations:** 1 Life Sciences Institute, Zhejiang University, 310058 Hangzhou, China; 2 Institute of Aging Research, Hangzhou Normal University, 311121 Hangzhou, China; 3 Fertility Preservation Laboratory, Reproductive Medicine Center, Guangdong Second Provincial General Hospital, 510317 Guangzhou, China; 4 Assisted Reproduction Unit, Department of Obstetrics and Gynecology, Sir Run Run Shaw Hospital, School of Medicine, Zhejiang University, Key Laboratory of Reproductive Dysfunction Management of Zhejiang Province, No. 3 Qingchun East Road, Jianggan District, 310016 Hangzhou, China

## Abstract

Trimethylation of histone H3 on lysine-4 (H3K4me3) is associated with gene-regulatory elements, but its transcription-independent function in cell division is unclear. CxxC-finger protein-1 (CFP1) is a major mediator of H3K4 trimethylation in mouse oocytes. Here we report that oocyte-specific knockout of *Cxxc1*, inhibition of CFP1 function, or abrogation of H3K4 methylation in oocytes each causes a delay of meiotic resumption as well as metaphase I arrest owing to defective spindle assembly and chromosome misalignment. These phenomena are partially attributed to insufficient phosphorylation of histone H3 at threonine-3. CDK1 triggers cell division–coupled degradation and inhibitory phosphorylation of CFP1. Preventing CFP1 degradation and phosphorylation causes CFP1 accumulation on chromosomes and impairs meiotic maturation and preimplantation embryo development. Therefore, CFP1-mediated H3K4 trimethylation provides 3a permission signal for the G2–M transition. Dual inhibition of CFP1 removes the SETD1–CFP1 complex from chromatin and ensures appropriate chromosome configuration changes during meiosis and mitosis.

## Abstract (web summary)

The transcription-independent function of trimethylation of histone H3 (H3K4me) in cell division is unclear. Here, Heng-Yu Fan and colleagues report that CFP1, a subunit of the H3K4 methyltransferase, is required for oocyte meiosis, being phosphorylated and degraded during cell cycle transition.

## Introduction

Histone posttranslational modifications (PTMs)—and specific combinations they create—mediate a wide range of nuclear events. Methylation of histone H3 on lysine-4 (H3K4me) is evolutionarily conserved from yeast to humans and is widely associated with gene-regulatory elements^1^. Particularly, the trimethylated species (H3K4me3), which is found primarily in active promoters, is implicated in transcriptional regulation^2^. In contrast to lysine methylation, phosphorylation of serine and threonine residues in histone H3 fluctuates substantially throughout the cell cycle, rising sharply when cells enter mitosis^3^. Histone H3 phosphorylation at threonine-3 (hereafter referred to as H3T3ph) is carried out by the kinase haspin early in prophase^4^. The close proximity of residues T3 and K4 in histone H3 potentially enables functional and regulatory interplay between the instances of phosphorylation and methylation^5,6^. Nonetheless, the possible direct function of H3K4me3 in cell division has been elusive because deficiency in this histone modification in cultured cells affects transcription of a broad spectrum of genes and causes cell cycle arrest in the G1 or S phase^7,8^. In this respect, a fully grown mammalian oocyte is an ideal model for studies on transcription-independent functioning of histone modifications because transcription is neither active nor required for the two sequential meiotic divisions^9,10^.

An oocyte-specific knockout of one of the genes coding for histone H3K4 methyltransferases or demethylases leads to impaired meiotic cell cycle progression, including delayed germinal vesicle breakdown (GVBD), distorted spindle assembly, error-prone chromosome separation, and compromised polar-body emission rates^11–13^. It has been proposed that overexpression or underexpression of certain key cell cycle regulators is responsible for these meiosis-related phenotypes^14,15^. Nevertheless, the evidence has been indirect and weak. The transcriptomes of fully grown GV oocytes are only moderately affected by epigenetic factors because their genome is transcriptionally quiescent^16^. In addition, epigenetic regulation of transcription is genome-wide. It is unlikely that genes of certain cell cycle regulators are specifically targeted in growing oocytes. In contrast, we hypothesized that multiple methylation of histone H3 at lysine-4 directly drives oocyte meiotic progression by regulating chromosome behaviors.

There are six major histone methyltransferases that catalyze H3K4 methylation: SETD1A, SETD1B, MLL1, MLL2, MLL3, and MLL4, which are subdivided into three distinct groups according to their sequence similarity and complex formation: SETD1A/B, MLL1/2, and MLL3/4^17,18^. In most cell types, SETD1-based complexes are the predominant H3K4 methyltransferases^19,20^. The SETD1 complex is driven to trimethylate H3K4 via its essential subunit CxxC-finger protein 1 (CFP1, encoded by the *Cxxc1* gene in mammals), which engages in multivalent chromatin binding to recognize both nonmethylated DNA and existing H3K4me3^21,22^. Zygotic knockouts of *Setd1a*, *Setd1b*, or *Cxxc1* in mice each results in early embryonic mortality due to a failure in gastrulation^23,24^. These pleiotropic phenomena illustrate fundamental roles for the SETD1–CFP1 complex in mammalian development but, at the same time, prevent detailed studies of its functions in adult cell types, including oocytes.

Recently, we generated an oocyte-specific *Cxxc1* knockout mouse strain using transgene *Zp3-Cre*, which is selectively expressed in growing oocytes, and demonstrated that SETD1–CFP1 is the major complex that mediates H3K4me3 accumulation in mouse oocytes^16^. Physiologically, maternal CFP1 and H3K4me3 are essential for the establishment of zygotic developmental competence. Meanwhile, spindle assembly was partially impaired in some CFP1-deleted oocytes that underwent meiotic maturation. Nevertheless, the oocyte transcriptome was only mildly affected in the CFP1-null oocytes; there was no sign of oocyte death and premature ovarian insufficiency in these mice before 6 months of age^16^. A conditional knockout of *Setd1b* in oocytes also caused oocyte maturation defects and female infertility, but the mechanism was investigated insufficiently^25^. We thus speculate that the SETD1–CFP1 complex and H3K4me3 may be involved in the regulation of meiotic cell division through a previously unidentified transcription-independent mechanism.

Given the fundamental role that the SETD1–CFP1 complex plays in depositing maternal H3K4me3 and sustaining female fertility, we investigate the transcription-independent function of CFP1 and its regulation during oocyte meiotic maturation. In contrast to our previous study in which *Cxxc1* was selectively knocked out in growing oocytes by *Zp3-Cre*, *Gdf9-Cre* was employed in the current investigation to knock *Cxxc1* out in oocytes as early as the primordial follicle stage, thereby giving the oocytes a longer time window to remove the existing CFP1 mRNA and protein after the gene knockout. We demonstrate that CFP1-mediated H3K4me3 accumulation in fully grown oocytes is a priming signal for meiotic cell cycle progression, particularly for T3 phosphorylation of histone H3. Moreover, we find that activity of the SETD1–CFP1 complex is dually inhibited by CDK1-triggered CFP1 phosphorylation and degradation at the onset of G2–M transition. Thus the downregulation of H3K4me3 and removal of CFP1 from chromatin are crucial for proper chromatin structure that is required for successful spindle assembly, accurate chromosome separation, and proper meiotic cell cycle progression.

## Results

### Dynamic histone H3K4 trimethylation and CFP1 accumulation in cell cycles

**Fig. 1:**
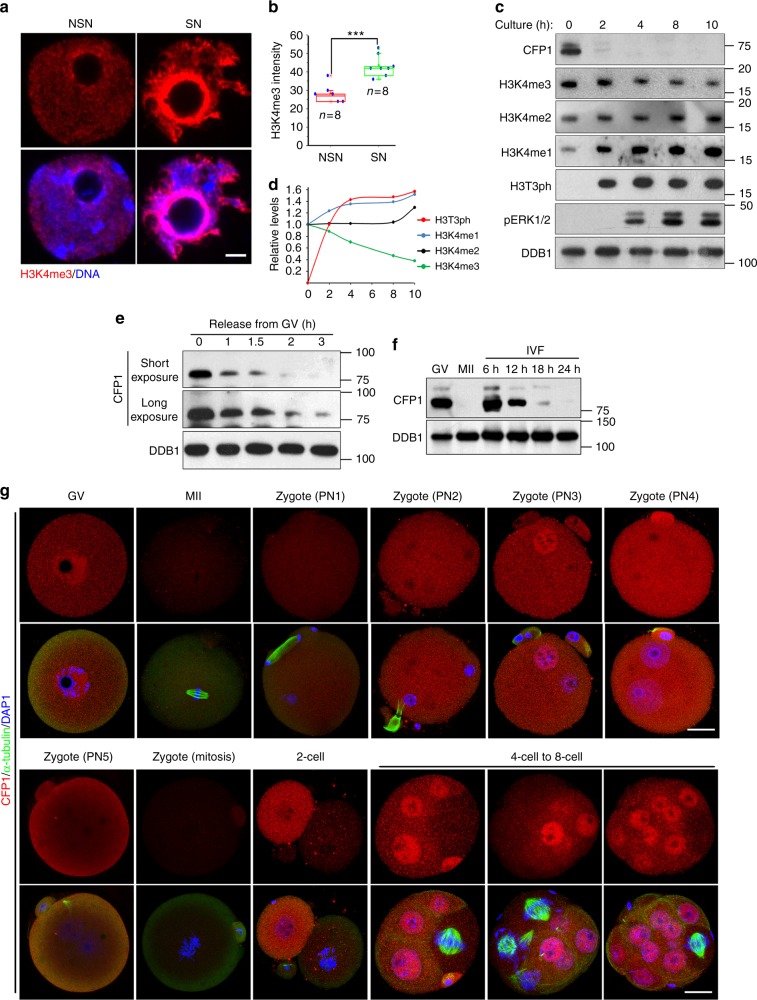
Fluctuation of CFP1 protein levels in oocyte meiosis and zygotic cleavages. **a** Immunofluorescence of trimethylated histone H3K4 (H3K4me3) on chromatin spreads made from postnatal day-21 mouse oocytes, which contained both non-surrounded nucleolus (NSN) and surrounded nucleolus (SN) type of chromatin configuration. More than eight oocytes of each type were observed with similar results. Scale bar, 5 μm. **b** Quantification of the H3K4me3 signal in (**a**). Numbers (*n*) of oocytes being quantified at each developmental stage are indicated. Error bars, S.E.M. ***P < 0.001 by two-tailed Student’s *t* tests. **c** Western blot results showing levels of the indicated proteins during oocyte in vitro maturation process. The constitutively expressed DDB1 was blotted as a loading control. Total proteins from 100 oocytes were loaded in each lane. **d** Relative levels of the indicated histone modifications normalized to the internal control (DDB1), corresponding to the results in (**c**). **e**, **f** Western blot results showing CFP1 levels during G2-M transition in maturing mouse oocytes (**e**) and the first mitosis in fertilized eggs (**f**). IVF in vitro fertilization. **g** CFP1 immunofluorescence results in mouse oocytes, zygotes, and preimplantation embryos. α-tubulin was co-stained to indicate dividing cells. Scale bars, 20 μm

In wild-type (WT) oocytes, chromatin-bound H3K4me3 levels increased during transition of chromatin configurations from the non-surrounded nucleolus (NSN) to surrounded nucleolus (SN) type, as determined by immunofluorescence analysis of GV chromatin spreads (Fig. 1a, b). During meiotic maturation, H3K4me3 levels gradually decreased after GVBD and reached the lowest point in anaphase I (Fig. 1c, d). In contrast, H3K4me1 and H3K4me2 levels increased during meiotic maturation (Fig. 1c, d). The meiotic maturation-coupled fluctuation of H3K4me3 levels suggested that this type of histone modification may perform a direct function in regulating meiotic cell cycle progression of oocytes.

It is noteworthy that the CFP1 protein level underwent a dramatic decrease during the G2–M transition and remained undetectable in maturing oocytes (Fig. 1c). Western blotting of oocyte samples collected at closer time points showed that CFP1 protein level quickly decreased within 1–2 h after oocytes were released from the GV arrest (Fig. 1e) and CFP1 remained undetectable in metaphase II (Fig. 1f). The CFP1 protein re-accumulated in zygotes after fertilization throughout the time period in which the fertilized eggs exited meiosis and entered interphase (Fig. 1f, g). Upon entering the first mitosis, CFP1 was degraded again (Fig. 1f, g). Furthermore, immunofluorescence results in 2–8-cell embryos revealed that CFP1 was degraded in each blastomere when it entered the second, third, and fourth mitosis (Fig. 1g).

**Fig. 2:**
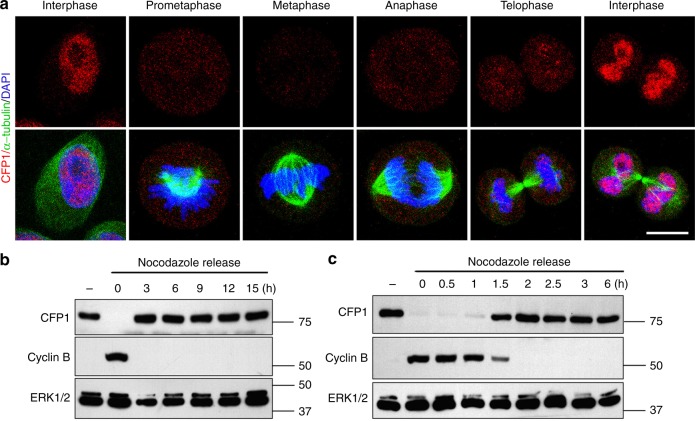
CFP1 degradation in mitotic HeLa cells. **a** CFP1 immunofluorescence results in HeLa cells at the indicated cell cycle stage. Scale bar, 5 μm. **b**, **c** CFP1 levels in cell cycle-synchronized HeLa cells. HeLa cells were arrested at metaphase by nocodazole (1 μM). Samples were taken at the indicated time points after being released from nocodazole arrest. Cyclin B1 was blotted as a marker of metaphase

In HeLa cells, CFP1 was detected in the nucleus of interphase cells but not in the cells that underwent mitotic divisions (Fig. 2a). In HeLa cells in which the cell cycle was arrested in metaphase by the microtubule disruptor nocodazole, the CFP1 protein was undetectable (Fig. 2b, c). When these cells were released from nocodazole treatment, CFP1 reappeared, accompanying the mitotic exit (Fig. 2b, c). Taken together, these results indicated that the CFP1 protein is transiently degraded during the meiotic and mitotic division. The observation of cell division–coupled CFP1 degradation inspired us to study the function of CFP1 and regulation of protein stability in oocytes and preimplantation embryos.

### CFP1 is crucial for spindle assembly and chromosome in murine oocyte

**Fig. 3:**
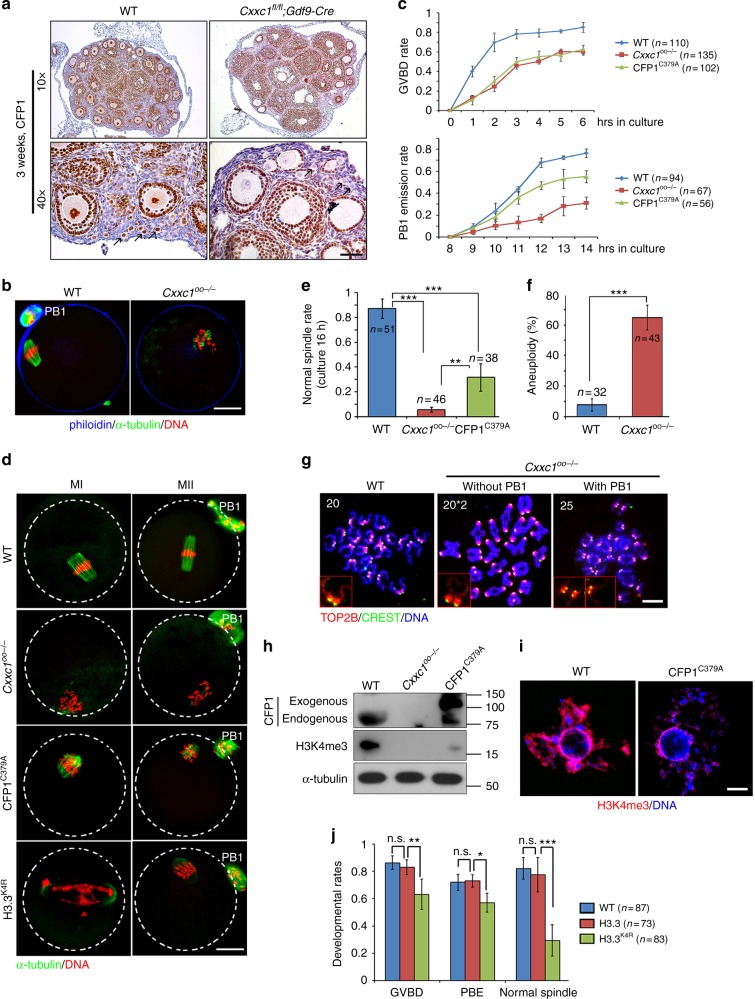
Oocyte-specific *Cxxc1* knockout causes meiotic maturation defects. **a** CFP1 immunohistochemistry on ovarian sections prepared from 3-week-old WT and *Cxxc1*^*fl/fl*^;*Gdf9-Cre* mice. Scale bar, 50 μm. **b** Confocal microscopic results of oocytes collected from oviducts of WT and *Cxxc1*^*oo−/−*^ mice, at 16 h after hCG injection. PB1 polar body-1. Scale bar, 20 μm. **c** Rates of germinal vesicle breakdown (GVBD) and PB1 emission in oocytes cultured in vitro. Fully grown GV oocytes were collected from PMSG-primed (44 h) PD23 mice of the indicated genotypes. Error bars, S.E.M. **d** Confocal microscopic results showing spindle assembly in cultured oocytes at metaphase I (MI) and metaphase II (MII). Scale bar, 20 μm. **e** Rates of normal spindle assembly in MI oocytes. Error bars, S.E.M. **P < 0.001 and **P < 0.01 by two-tailed Student’s *t* tests. **f** Rates of MII oocytes containing abnormal numbers of chromosomes. Error bars, S.E.M. ***P < 0.001 by two-tailed Student’s *t* tests. **g** Representative immunofluorescence images of chromosome spreads made from WT and *Cxxc1*^*oo−/−*^ oocytes after 18 h of in vitro maturation culture. Numbers of chromosome pairs are indicated. Scale bar, 5 μm. **h**, **i** Western blot (**h**) and immunofluorescence (**i**) results showing CFP1 and H3K4me3 levels in WT and *Cxxc1*^*oo−/−*^ oocytes as well as WT oocytes overexpressing CFP1^C379A^ for 12 h in vitro. Scale bar, 5 μm. **j** Rates of GVBD, PB1 emission, and normal spindle assembly in oocytes cultured in vitro. Fully grown GV oocytes were microinjected with mRNAs encoding WT and K4-mutated histone H3.3 and were released to resume meiotic maturation at 12 h after microinjection. Error bars, S.E.M. ***P < 0.001, **P < 0.01, and *P < 0.05 by two-tailed Student’s *t* tests. n.s., non-significant

CFP1 is expressed in the nucleus of oocytes as early as the primordial follicle stage (Fig. 3a). To study the function of CFP1 in oocyte maturation, we specifically knocked out *Cxxc1* in mouse oocytes as early as the primordial follicle stage using the *Gdf9-Cre*. Efficient CFP1 protein depletion in oocytes was confirmed by immunohistochemistry (Fig. 3a). *Cxxc1*^*fl/fl*^;*Gdf9-Cre* female mice (hereafter referred to as *Cxxc1*^*oo–/–*^) are completely infertile. Nonetheless, the CFP1-null oocytes were able to develop to the fully grown GV stage (Fig. 3a and Supplementary Fig. 1a) and could be ovulated by superovulation treatment (Supplementary Fig. 1b). We collected these oocytes from oviducts and analyzed their potential defects in oocyte meiotic maturation, at 16 h after human chorionic gonadotropin (hCG) injection. Immunofluorescence and confocal microscopic results showed that the majority of ovulated CFP1-null oocytes contained distorted spindles and chromosomes were not properly aligned at the equatorial plate (Fig. 3b). To examine the meiotic maturation process more closely, we isolated fully grown GV oocytes from pregnant mare serum gonadotropin (PMSG)-primed *Cxxc1*^*oo–/–*^ mice and cultured them in vitro. These oocytes resumed meiosis more slowly than WT oocytes did and manifested reduced GVBD and polar body 1 (PB1) emission rates (Fig. 3c). Similar to those that underwent meiotic maturation in vivo, the in vitro-matured CFP1-null oocytes failed to assemble bipolar spindles, and chromosomes were not aligned at the equatorial plates at both the MI and MII stages (Fig. 3d). We observed that 30% of CFP1-deleted oocytes released PB1s and developed to metaphase II (MII), but the spindles were distorted (Fig. 3d, e). In addition, their chromosomes were mostly aneuploid (Fig. 3f), as counted in chromosome spreads (Fig. 3g).

In *Cxxc1*^*fl/fl*^;*Gdf9-Cre* mice, CFP1 was deleted in oocytes as early as at the primordial follicle stage (Fig. 3a). Therefore, the defects of meiotic maturation in CFP1-null oocytes might have been caused by some indirect and nonspecific factors accumulated during oocyte growth. To test whether CFP1 is directly involved in oocyte meiotic maturation, we expressed a dominant-negative CFP1 mutant (CFP1^C379A^) in fully grown GV oocytes by mRNA microinjection. This mutant protein can bind to DNA but fails to recruit SETD1, and therefore has a dominant-negative effect^26^. The expression of hemagglutinin (HA)-tagged CFP1^C379A^ at a level comparable with that of the endogenous CFP1 was detected by western blotting (Fig. 3h). In addition, western blotting and immunofluorescence results also indicated that the H3K4me3 level was significantly lower in CFP1-null and CFP1^C379A^-overexpressing oocytes (Fig. 3h, i). Notably, in the in vitro maturation experiment, the CFP1^C379A^-overexpressing oocytes had a low GVBD rate, similar to that of oocytes isolated from *Cxxc1*^*oo–/–*^ mice (Fig. 3c, upper panel). In addition, the PB1 emission rate in CFP1^C379A^-overexpressing oocytes was significantly lower than that of the control oocytes (Fig. 3c, lower panel). Furthermore, overexpression of CFP1^C379A^ impaired spindle formation at both stages MI and MII (Fig. 3d, e). Overall, overexpression of a dominant-negative CFP1 mutant in fully grown oocytes mimicked the phenotype of the oocyte-specific *Cxxc1* knockout. Considering the fact that transcriptional activity is absent in fully grown oocytes, our results indicated that CFP1 has a transcription-independent role in the regulation of oocyte meiotic maturation.

To obtain more direct evidence, we ectopically expressed the H3.3^K4R^ mutant protein in GV oocytes by mRNA microinjection. According to other studies, H3.3 is the major H3 variant that undergoes K4 trimethylation in mouse oocytes^27,28^. This H3.3^K4R^ mutant deficient for methylation was expressed and efficiently incorporated into chromatin (Supplementary Fig. 2a). As predicted, the chromatin-bound H3K4me3 amount was low in oocytes expressing Flag-H3.3^K4R^ but not in those expressing exogenous Flag-H3.3^WT^ (Supplementary Fig. 2a). Furthermore, the H3.3^K4R^-expressing oocytes had meiosis defects resembling to those of CFP1-deleted oocytes: GVBD and PB1 emission rates decreased (Fig. 3j); and spindle assembly and equatorial chromosome alignment failed (Fig. 3d, bottom panels; and Fig. 3j). These results indicated that methylation of histone H3 at K4 is a prerequisite for successful meiotic maturation.

### CFP1 deletion in oocytes caused meiotic cell cycle arrest in metaphase I

**Fig. 4:**
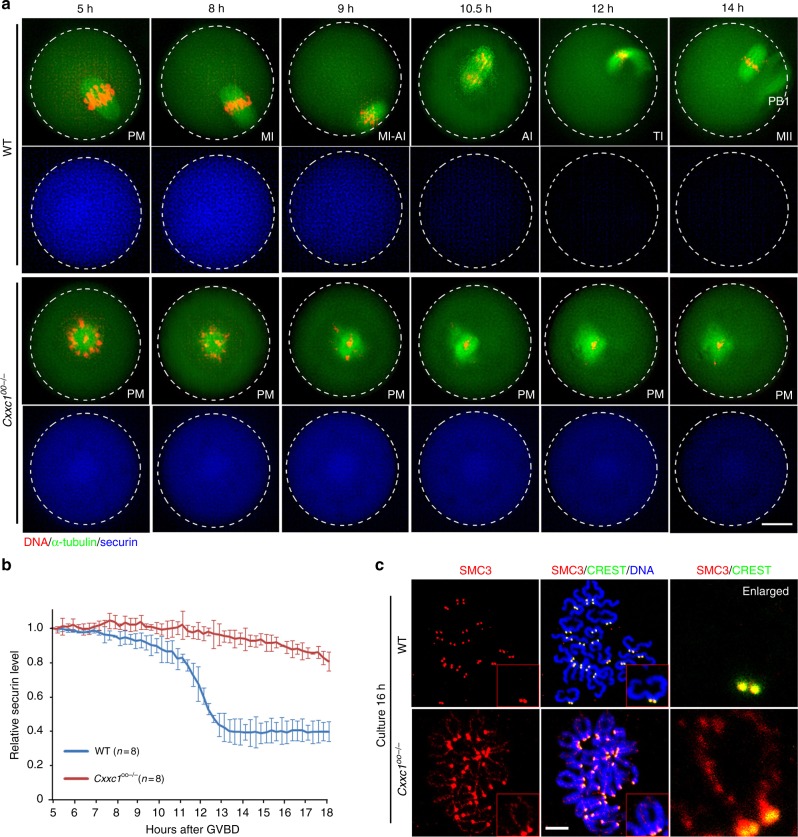
Meiotic cell cycle progression in WT and *Cxxc1*^*oo−/−*^ oocytes. **a** Live imaging results showing in vitro meiotic division of WT and *Cxxc1*^*oo−/−*^ oocytes. Pre-MI pre-metaphase, MI metaphase I, MI-AI metaphase–anaphase transition, AI anaphase I, AI-MII anaphase I–metaphase II transition, MII metaphase II. Scale bar, 20 μm. **b** Data represent the mean and standard deviations of mCherry-securin fluorescence intensity levels in WT (blue) and *Cxxc1*^*oo−/−*^ (red) oocytes at each time point. Time after GVBD is indicated. Values from individual oocytes were normalized relative to that at 5 h. Error bars, S.E.M. **c** SMC3 immunofluorescence (red) showing cohesin on chromosomes of WT and *Cxxc1*^*oo−/−*^ oocytes at 16 h after in vitro culture. Centromeres were labeled by CREST staining (green). DNA was labeled by DAPI (blue). Scale bar, 5 μm

Employing live cell imaging microscopy, we next compared the dynamic spindle assembly and meiotic cell cycle progression between WT and CFP1-deleted oocytes. GV-arrested oocytes were microinjected with mRNA encoding green fluorescent protein (GFP)-tagged α-tubulin (to label spindles) and were cultured in a medium containing Hoechst-33342 (to label DNA). Oocytes that underwent GVBD within 4 h after being released from milrinone were subjected to live cell imaging. Results in Fig. 4a reveal that WT oocytes finished meiosis I and developed to MII at the end of cultivation. In contrast, bipolar spindle formation, equatorial chromosome alignment, and PB1 emission failed in *Cxxc1*^*oo−/−*^ oocytes.

To assess the kinetics of metaphase-to-anaphase transition, we microinjected mRNA encoding mCherry-securin into WT and *Cxxc1*^*oo−/−*^ oocytes and monitored the dynamics of mCherry fluorescence. As shown in Fig. 4a and quantified in Fig. 4b, securin was degraded in WT oocytes approximately 10–12 h after meiotic resumption but remained stable in CFP1-deleted oocytes up to 18 h after meiotic resumption, suggesting that the CFP1-deleted oocytes failed to enter anaphase I.

At the end of in vitro maturation culture, oocytes were collected for preparing chromosome spreads. Immunofluorescence results in Fig. 4c show that all WT oocytes under study (*n* = 26) contained 20 pairs of coherent sister chromatids, and the cohesion core subunit SMC3 was detected only at the kinetochore (labeled with CREST antibody). On the other hand, most *Cxxc1*^*oo−/−*^ oocytes (21 out of 32) contained unseparated homologous chromosomes with remarkable SMC3 distribution on chromosome arms. Thus, in CFP1-null oocytes, the anaphase-promoting complex, which removes securin, and the separase, which in turn removes cohesin, were not activated, and the meiotic cell cycle was arrested at pre-MI.

### CFP1-dependent H3K4me3 accumulation is a prerequisite for H3T3 phosphorylation

**Fig. 5:**
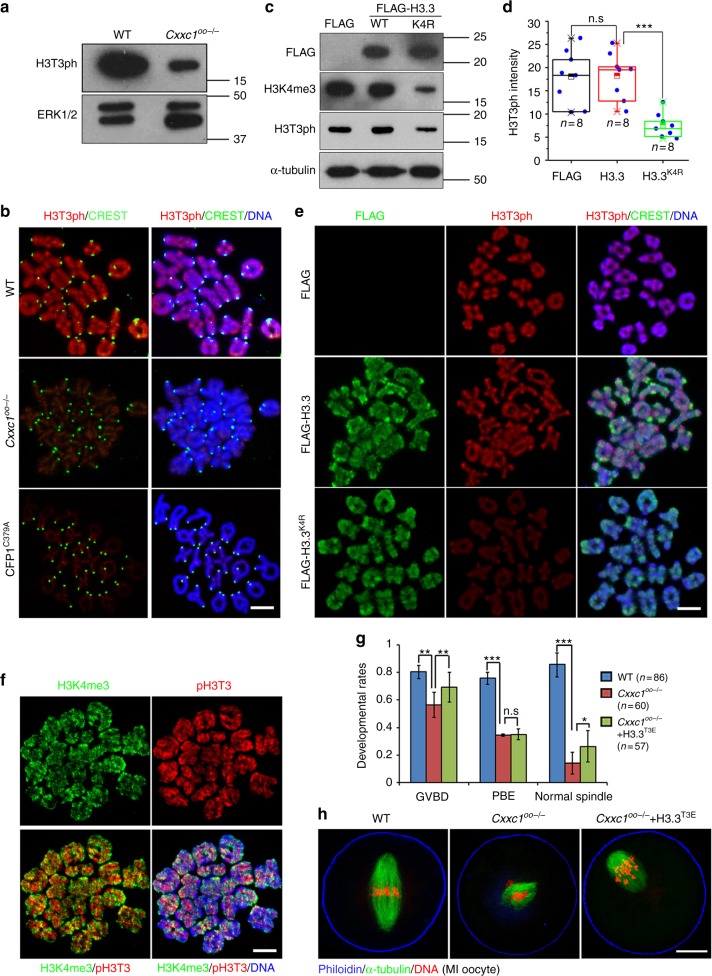
H3K4 trimethylation is a prerequisite for H3T3 phosphorylation and meiotic cell cycle progression. **a** Western blot of H3T3ph in WT and *Cxxc1*^*oo−/−*^ oocyte at 6 h after in vitro culture. **b** Immunofluorescence of H3T3ph on WT and *Cxxc1*^*oo−/−*^ oocyte chromosome spreads prepared at MI stage. Centromeres and DNA were labeled by CREST immunofluorescence (green) and DAPI staining (blue), respectively. Scale bar, 5 μm. **c** Western blot of the indicated proteins in WT oocytes ectopically express Flag-tagged histone H3.3^WT^ and H3.3K4^K4R^. **d** Quantification of H3T3ph signals in (**e**). Error bars, S.E.M. ***P < 0.001 by two-tailed Student’s *t* tests. n.s., non-significant. **e** Immunofluorescence of Flag-tagged histone H3.3 (H3.3^WT^ and H3.3K4^K4R^) and H3T3ph on chromosome spreads prepared at MI stage. Fully grown WT oocytes at the GV stage were microinjected with mRNAs encoding histone H3.3^WT^ or H3.3^K4R^ and were released to resume meiotic maturation at 12 h after microinjection. Scale bar, 5 μm. **f** Co-immunofluorescence of H3K4me3 and H3T3ph on MI chromosome spreads prepared from WT oocytes. Scale bar, 5 μm. **g** Rates of GVBD, PB1 emission, and normal spindle assembly in oocytes cultured in vitro. Fully grown WT oocytes at the GV stage were microinjected with mRNAs encoding histone H3.3^WT^ or H3.3^T3E^ and were released to resume meiotic maturation at 12 h after microinjection. Error bars, S.E.M. ***P < 0.001, **P < 0.01, and *P < 0.05 by two-tailed Student’s *t* tests. n.s., non-significant. **h** Immunofluorescent staining of α-tubulin showing spindle assembly in WT oocytes microinjected with mRNAs encoding Flag-tagged histone H3.3^WT^ or H3.3^T3E^. Scale bar, 20 μm

Phosphorylation of histone H3 at threonine-3 during the G2–M transition is required for oocyte meiotic maturation^3^. T3 of histone H3 is promptly phosphorylated during the G2–M transition. High H3T3ph levels were maintained throughout meiosis I (Fig. 1c, d). Blocking this K4-adjacent H3 modification causes similar defects of oocyte maturation as we observed in *Cxxc1*^*oo−/−*^ oocytes^3^. Therefore, we hypothesized that CFP1-dependent H3K4 trimethylation is a priming signal for H3T3 phosphorylation. Consistent with this hypothesis, the H3T3ph level was significantly compromised in *Cxxc1*-null oocytes at 6 h after in vitro culture (Fig. 5a). Similarly, overexpression of the dominant-negative CFP1 (CFP1^C379A^) also blocked H3T3 phosphorylation on chromosomes (Fig. 5b). In contrast, phosphorylation of histone H3 at Ser-10 (H3S10ph), another cell division–related histone H3 modification, was not affected in *Cxxc1*^*oo−/−*^ oocytes (Supplementary Fig. 2b, c). This result indicated that the role of CFP1 in regulating H3T3ph is specific.

More directly, when the oocytes overexpressing H3.3^K4R^ were allowed to undergo meiotic maturation, total and chromosomal H3T3ph levels also decreased (Fig. 5c–e). The H3T3ph western blot and immunofluorescence results in Fig. 5b, c, e represent only endogenous protein because the anti-H3T3ph antibody does not recognize any K4-mutation-bearing H3 forms, due to the close proximity of these mutations to the T3ph epitope. A co-immunofluorescence assay of H3T3ph and H3K4me3 in MI oocytic chromosome spreads (using a rabbit- and a mouse-derived antibody, respectively) indicated that these two types of histone H3 modifications did not overlap strongly (Fig. 5f). Rather, H3T3ph preferentially accumulated at the interaction interface between homologous chromosomes, whereas H3K4me3 tended to be located at the surface of paired chromosomes (Fig. 5f). Collectively, these results strongly indicated that methylated H3K4 regulates T3 phosphorylation in an intermolecular manner.

### Histone H3T3 phosphorylation partially reverses the defects in CFP1-null oocytes

The evidence so far indicated that CFP1-mediated H3K4 trimethylation is upstream of H3T3 phosphorylation in the control over meiotic progression. Therefore, we determined whether a histone H3.3 mutant that mimics T3 phosphorylation (H3.3^T3E^) can partially reverse the meiosis defects in CFP1-null oocytes. This histone mutant was expressed in GV oocytes of WT and *Cxxc1*^*oo−/−*^ mice by mRNA microinjection. After being released from the GV arrest, the H3.3^T3E^ expressing CFP1-null oocytes has a GVBD rate close to that of normal oocytes (Fig. 5g). Although the PB1 emission rate was not rescued by H3.3^T3E^, the spindles were assembled with better morphology than in CFP1-null oocytes expressing H3.3^T3E^ (Fig. 5g, h). These results suggested that an important function of CFP1-mediated H3K4 trimethylation is to facilitate H3T3 phosphorylation and that these hierarchical histone modifications are crucial for meiotic cell cycle progression.

### CDK1 triggers CFP1 phosphorylation and degradation

The results so far indicated that CFP1-dependent H3K4me3 accumulation in fully grown GV oocytes is a priming signal for proper meiotic cell cycle progression. In contrast, we also observed prompt CFP1 degradation during the G2–M transition in both oocytes and early blastomeres. Therefore, we further investigated the physiological importance and biochemical mechanism of CFP1 removal during meiotic and mitotic divisions.

**Fig. 6:**
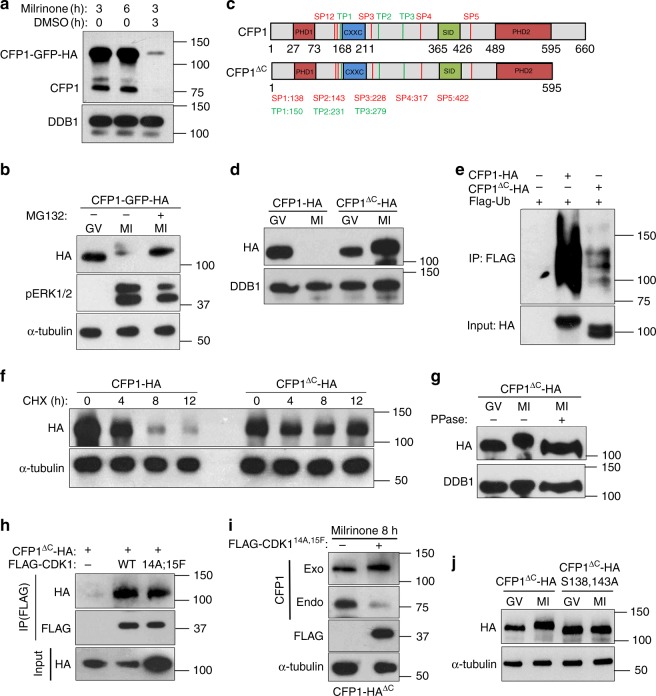
CFP1 proteins are degraded during G2-M transition in oocytes. **a** CFP1 degradation during oocyte maturation. GV oocytes were microinjected with mRNA encoding CFP1-GFP-HA and cultured in medium containing milrinone. Some oocytes were removed from milrinone-containing medium 3 h later and further cultured for another 3 h in milrinone-free medium. Total proteins from 100 oocytes were loaded in each lane. **b** MG132 blocked CFP1 degradation in maturing oocytes. GV oocytes were microinjected with mRNA encoding CFP1-GFP-HA and cultured in medium containing milrinone for 3 h and then were transferred to milrinone-free medium with or without MG132 for further culture (6 h). **c** Functional domains and potential CDK1 phosphorylation sites (S/T-P) of mouse CFP1. **d** The C-terminus-deleted CFP1 (CFP1^ΔC^) was not degraded in maturing oocytes. GV oocytes were microinjected with mRNA encoding CFP1-HA or CFP1^ΔC^-HA and cultured as in (**b**). **e** Ubiquitination levels of CFP1-HA or CFP1^ΔC^-HA in HeLa cells. Cells were co-transfected with plasmids expressing the indicated proteins. Ubiquitin (Ub)-bound proteins were pulled-down from cell lysates at 48 h after transfection with Flag-affinity agarose beads. **f** Cycloheximide (CHX) chasing experiment showing CFP1 stability. HeLa cells were transfected with plasmids expressing CFP1-HA or CFP1^ΔC^-HA for 48 h and then incubated with medium containing CHX (10 μM). Cells were lysed at 0, 4, 8, and 12 h after CHX treatment and subjected to western blot. **g** Band shift showing CFP1 phosphorylation. GV oocytes were microinjected and cultured as in (b). Some samples were pre-incubated with protein phosphatase for 2 h before western blot. **h** Co-IP results showing the interaction between CFP1 and CDK1. HeLa cells were co-transfected with plasmids expressing CFP1^ΔC^-HA and CDK1 for 48 h before immunoprecipitation. **i** Constitutively active CDK1 triggers CFP1 phosphorylation and degradation. GV oocytes were microinjected with mRNAs encoding CFP1^ΔC^-HA and Flag-CDK1^14A;14F^ and cultured in medium containing milrinone for 8 h. **j** Mutation of S138 and S143 abolished the CFP1 band shift in maturing oocytes. GV oocytes were microinjected with mRNAs encoding indicated CFP1 forms and cultured as in (**b**)

To easily monitor CFP1 degradation in oocytes, we constructed a plasmid expressing a CFP1-GFP-HA fusion protein. After in vitro transcription (IVT) and microinjection of the mRNA into GV oocytes, the CFP1-GFP-HA fusion protein was abundantly expressed. Just as endogenous CFP1, this fusion protein was degraded within 3 h of meiotic resumption (Fig. 6a). Furthermore, its degradation was prevented by addition of the 26S proteasome inhibitor MG132 into the culture medium, suggesting that CFP1 was degraded via the canonical ubiquitin–proteasome pathway (Fig. 6b). After a series of domain-mapping analyses, we found that deleting the C-terminal 65 amino acids (aa) stabilized CFP1 in maturing oocytes (Fig. 6c, d). In HeLa cells transfected with a CFP1-expressing plasmid, deletion of the C-terminal 65 aa significantly inhibited the polyubiquitination of CFP1 (Fig. 6e) and prolonged half-life of the CFP1 protein when protein synthesis was inhibited by cycloheximide treatment (Fig. 6f). Moreover, we noticed an upshift of the CFP1 band on a gel (after sodium dodecyl sulfate-polyacrylamide gel electrophoresis (SDS-PAGE)), specifically in MI oocytes, when CFP1 degradation was blocked by MG132 treatment (Fig. 6b) or C-terminal deletion (Fig. 6d). This band upshift was abrogated when the protein sample was pretreated with protein phosphatase (Fig. 6g), indicating that the upshift was caused by CFP1 phosphorylation.

Based on this observation, we attempted to identify the protein kinase that mediates meiotic maturation–coupled CFP1 phosphorylation and degradation. In a co-immunoprecipitation (co-IP) experiment, CFP1^ΔC^ interacted with CDK1, the major kinase that triggers the G2–M transition in the cell cycle (Fig. 6h). We generated constitutively active CDK1, in which the inhibitory phosphorylation sites (Thr-14 and Tyr-15) were mutated to alanine and phenylalanine, respectively. This CDK1 mutant (CDK1^14A,15F^) bound to CFP1^ΔC^ as well (Fig. 6h). When we coexpressed CDK1^14A;15F^ and CFP1^ΔC^ in GV oocytes by mRNA microinjection, CDK1^14A;15F^ triggered premature degradation of endogenous CFP1 and phosphorylation of CFP1^ΔC^ (Fig. 6i). On the other hand, specific inhibition of haspin and aurora A, two kinases that also mediate the G2–M transition but downstream to CDK1, did not prevent the GVBD-associated CFP1 phosphorylation (Supplementary Fig. 3a, b). Taken together, these results strongly indicated that CDK1 directly interacts with CFP1 and phosphorylates it at the onset of the G2–M transition.

The CFP1 protein contains multiple consensus CDK1 phosphorylation sites (SP and TP) that are conserved among vertebrates (Fig. 6c). By a series of point mutagenesis analyses, we found that mutation of two SP sites (Ser-138 and Ser-143) close to, but not within, the CXXC finger domain of CFP1 completely blocked CFP1 phosphorylation in maturing oocytes (Fig. 6j). Nevertheless, the full-length CFP1 protein that bears these two point mutations (CFP1^2SA^) was degraded normally after meiotic resumption (Supplementary Fig. 3c). This result suggests that, although CDK1 triggers both phosphorylation and degradation of CFP1 protein, phosphorylation of CFP1 by CDK1 is not a prerequisite for its degradation during cell division. Therefore, CDK1 may trigger CFP1 degradation through some indirect mechanisms rather than CFP1 phosphorylation. The biochemical and cellular function of CFP1 phosphorylation was further addressed below.

### Preventing CFP1 degradation and phosphorylation impairs oocyte maturation

**Fig. 7:**
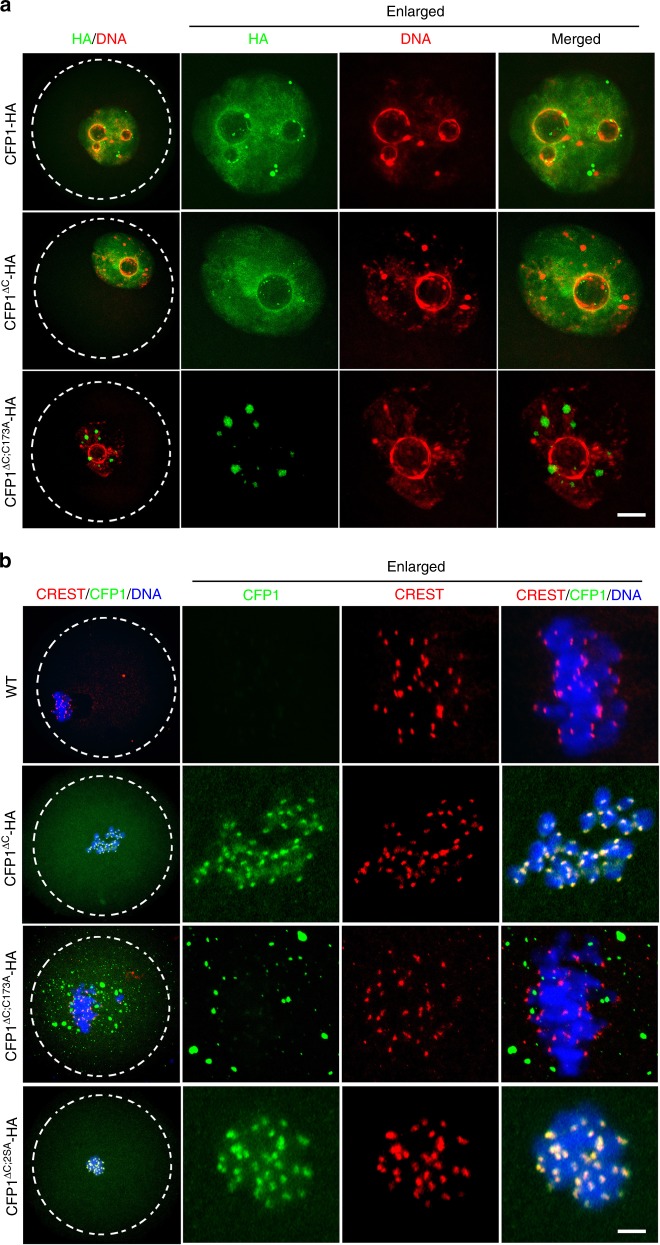
Localization of wild-type and mutated CFP1 forms in oocytes. **a**, **b** Immunofluorescence results showing localization of indicated CFP1 forms in oocytes at GV (**a**) and MI (**b**) stages. GV oocytes were microinjected with mRNAs encoding the indicated CFP1 forms and cultured in medium containing milrinone for 3 h, and then some of them were transferred to milrinone-free medium for further culture (6 h). Scale bar, 5 μm

To assess the cellular importance of CFP1 degradation, we microinjected mRNAs encoding WT and nondegradable CFP1 (CFP1^ΔC^ and CFP1^ΔC;2SA^) into GV oocytes and examined the process of their meiotic maturation. In addition, to test whether the potential function of the stabilized CFP1 depends on its DNA-binding ability, we generated a CFP1 mutation in which a key cysteine residue required for DNA binding (C173)^29^ was mutated to alanine in the CFP1^ΔC^ background (CFP1^ΔC;C173A^). In GV oocytes, both CFP1^WT^ and CFP1^ΔC^ were relatively ubiquitously distributed in the nucleus except for the nucleolus (Fig. 7a). CFP1^ΔC;C173A^ was also located in the nucleus but formed multiple foci that did not overlap with DNA (Fig. 7a). When the microinjected oocytes developed to the MI stage, CFP1^WT^ was degraded and became undetectable (Fig. 7b). In contrast, CFP1^ΔC^ and CFP1^ΔC;2SA^ were stably expressed and enriched on chromosomes, particularly in the kinetochores (Fig. 7b). CFP1^ΔC;C173A^ was also stabilized but existed as large cytoplasmic particles without co-localization with chromosomes (Fig. 7b).

**Fig. 8:**
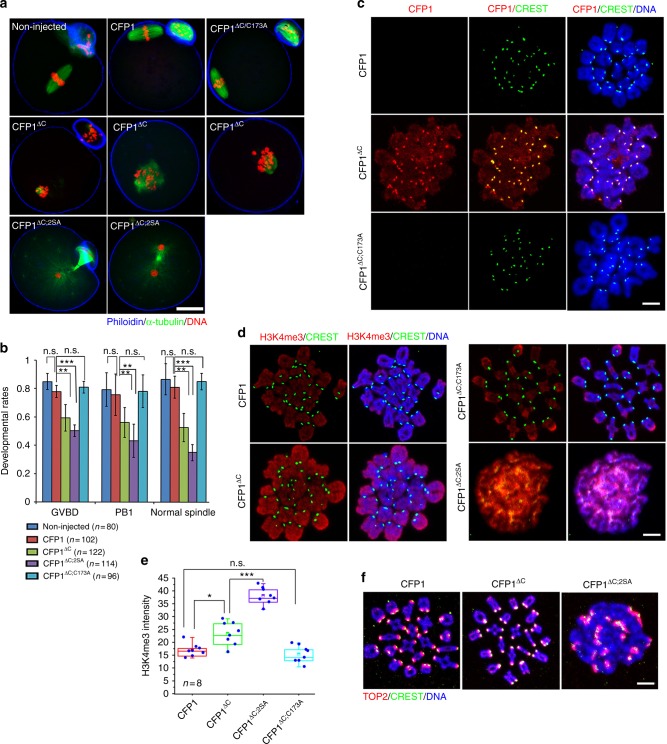
CFP1 degradation is required for appropriate histone H3 modifications and normal spindle assembly. **a** Immunofluorescent staining of α-tubulin showing spindle assembly in oocytes microinjected with mRNAs encoding WT or mutated CFP1. Oocyte subcortical actin microfilaments and DNA were labeled by phalloidin (red) and DAPI (blue), respectively. Scale bar, 20 μm. Fully grown GV oocytes from WT mice were used for mRNA microinjection throughout the experiments presented in this figure. **b** GVBD and PB1 emission rates in oocytes microinjected with mRNAs encoding CFP1 (WT and mutants), as well as the percentages of oocytes containing a normal spindle at MI stage. GV oocytes were microinjected with mRNAs encoding CFP1 (WT and mutants) and were allowed to resume meiotic maturation for 8 h. Error bars, S.E.M. ***P < 0.001 and **P < 0.01 by two-tailed Student’s *t* tests. n.s., non-significant. **c**, **d** Immunofluorescence of CFP1 (**c**) and H3K4me3 (**d**) on oocyte chromosome spreads prepared at MI stage. Centromeres and DNA were labeled by CREST immunofluorescence (green) and DAPI staining (blue), respectively. Scale bar, 5 μm. **e** Quantification of H3K4me3 fluorescent signals in (**d**). Error bars, S.E.M. ***P < 0.001, **P < 0.01, and *P < 0.05 by two-tailed Student’s *t* tests. n.s., non-significant. **f** Chromosome spreads made from MI oocytes overexpressing WT and mutated CFP1. Centromeres, chromosome arms, and DNA were labeled by CREST (green), Topoisomerase II (TOP2, red), and DAPI (blue) staining, respectively. Scale bar, 5 μm

Ectopic expression of CFP1^WT^ in GV oocytes did not affect meiotic maturation (Fig. 8a, b). This finding is conceivable because CFP1 was quickly degraded during the G2–M transition. On the other hand, both GVBD and PB1 emission rates decreased in oocytes overexpressing stabilized CFP1^ΔC^ (Fig. 8b). Confocal microscopy showed that spindle assembly was severely affected in these oocytes (Fig. 8a, middle panels, and Fig. 8b). Moreover, these phenotypes are remarkably strengthened in oocytes overexpressing CFP1^ΔC;2SA^ (Fig. 8a, bottom panels, and 8b). Although CFP1^ΔC;C173A^ was not degraded, it did not cause any abnormality during meiotic maturation (Fig. 8a, b), indicating that only the DNA-binding CFP1 is detrimental to meiotic progression of the oocyte.

We further examined the chromosome spreads made from MI oocytes. While the ectopically expressed CFP1^WT^ was degraded and not detected on chromosomes, CFP1^ΔC^ was present on the chromosome arms but, unexpectedly, was more concentrated in centromeres (Fig. 8c). Furthermore, the chromosomal H3K4me3 amount was high in oocytes expressing the degradation-resistant CFP1^ΔC^ (Fig. 8d, e). CFP1^ΔC;2SA^ overexpression not only caused a more significant increase in the H3K4me3 level than CFP1^ΔC^ did (Fig. 8d, e) but also impaired the condensation and organization of chromosomes: poorly condensed chromosomes formed a clump in oocytes and single chromosomes were difficult to distinguish (Fig. 8a bottom panels; Figs. 8d, f; 7b, bottom panels). In contrast, CFP1^ΔC;C173A^ was neither present on chromosomes (Fig. 8c) nor did it affect the H3K4me3 level in oocytes (Fig. 8d, e).

### Phosphorylation of CFP1 decreases its chromatin-binding ability

**Fig. 9:**
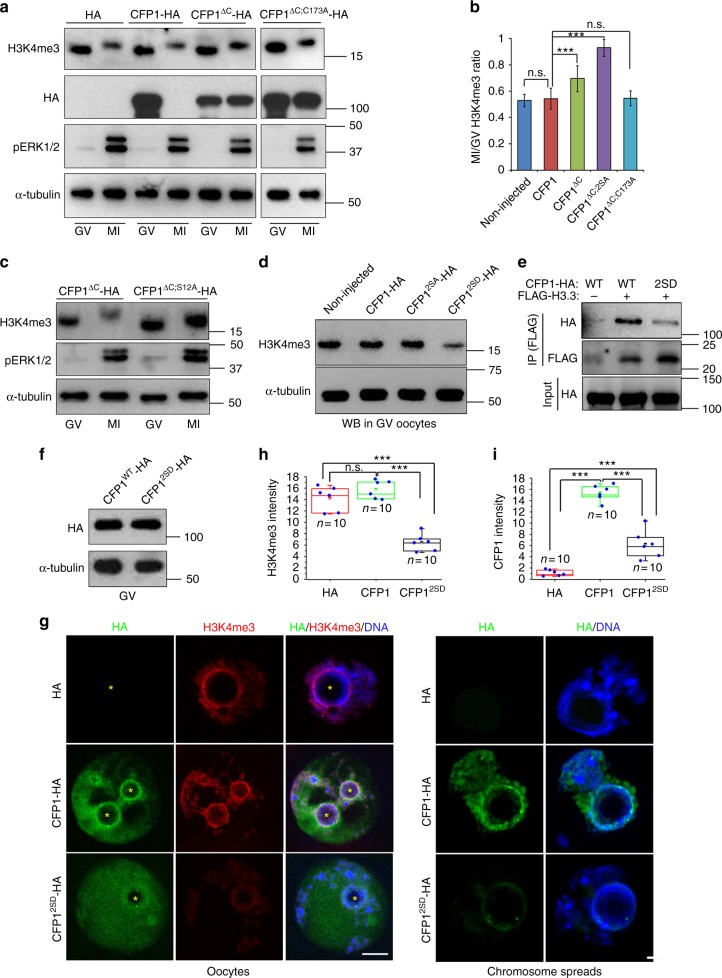
CFP1 function is inhibited by phosphorylation. **a** Western blots showing levels of HA-tagged CFP1 (WT and mutants) and H3K4me3 in microinjected oocytes, at 8 h after release into meiotic maturation. Phosphorylated ERK1/2 was blotted as a marker of meiotic resumption, and α-tubulin was blotted as a loading control. Fully grown GV oocytes from WT mice were used for mRNA microinjection throughout the experiments presented in this figure. **b** Ratio of H3K4me3 levels between MI and GV stages in oocytes overexpressing WT and mutated CFP1, based on western blot results in (**a**, **c**). **c** Western blots showing H3K4me3 levels in oocytes overexpressing indicated CFP1 mutants, at 8 h after release into meiotic maturation. **d** Western blot results showing H3K4me3 levels in GV oocytes overexpressing the indicated CFP1 mutants for 12 h after mRNA microinjection. **e** Co-IP results showing interaction between CFP1 and histone H3.3 in HeLa cells transfected with plasmids expressing the indicated recombinant proteins. **f** Expression levels of WT and phosphorylation site-mutated CFP1 in GV oocytes at 12 h after mRNA microinjection. **g** Immunofluorescence results showing CFP1 localization and H3K4me3 levels in the nuclei of GV oocytes after mRNA microinjection as in (**d**). Scale bar, 5 μm. Nucleoli are indicated by asterisks. **h**, **i** Quantification of H3K4me3 signals in **g**, **j**, respectively. Error bars, S.E.M. ***P < 0.001 by two-tailed Student’s *t* tests. n.s., non-significant. **j** Immunofluorescence on chromatin spread made from GV oocytes after mRNA microinjection as in **g**, showing levels of chromatin-bound CFP1. Scale bar, 5 μm

Western blotting analysis of whole-oocyte lysates confirmed that the H3K4me3 level increased at the MI stage in CFP1^ΔC^-expressing but not in CFP1^ΔC;C173A^-expressing oocytes (Fig. 9a, b). Although CFP1^ΔC^ was not degraded, it did not completely prevent the decrease of total H3K4me3 levels in MI oocytes (Fig. 9a–c). On the other hand, overexpression of phosphorylation site–mutated CFP1^ΔC^ (CFP1^ΔC;2SA^) was more effective than CFP1^ΔC^ at maintaining a high H3K4me3 level in MI oocytes (Fig. 9b, c). Moreover, we generated phosphorylation-mimicking mutations in CFP1^ΔC^ (Ser-138 and Ser-143 were mutated to aspartic acid, CFP1^ΔC;2SD^). When CFP1^ΔC;2SD^ was overexpressed in GV oocytes, it had a dominant-negative effect by decreasing the H3K4me3 level (Fig. 9d). Taken together, these results indicated that phosphorylation of CFP1 at Ser-138 and Ser-143 inhibits its ability to mediate H3K4 trimethylation, and this phenomenon is independent of CFP1 degradation.

We investigated the mechanism by which CFP1 phosphorylation inhibits H3K4me3 accumulation. In co-IP experiments, mutations of CFP1 phosphorylation sites did not affect CFP1 binding to SETD1 (Supplementary Fig. 3d) but reduced the interaction between CFP1 and histone H3 (Fig. 9e). We ectopically expressed CFP1^WT^ or CFP1^2SD^ in GV oocytes at comparable levels (Fig. 9f). CFP1^WT^ showed an uneven distribution pattern in the nucleus and was more concentrated in the chromatin area (Fig. 9g). On the other hand, CFP1^2SD^ was evenly distributed in the entire nucleus excluding the nucleolus. In addition, H3K4me3 levels slightly increased in oocytes expressing CFP1^WT^ but remarkably decreased in oocytes expressing CFP1^2SD^ (Fig. 9g, h). This observation is in agreement with the western blot results in Fig. 9d. Furthermore, we prepared chromatin spreads from these oocytes. With this procedure, free proteins and proteins loosely bound to chromatin in the nucleus were removed. Only proteins tightly bound to chromatin remained. Immunofluorescence results showed that CFP1^WT^ was detected in GV chromatin spreads, but the signal of CFP1^2SD^ was much weaker in comparison to CFP1^WT^ (Fig. 9l, j).

The identified CFP1 phosphorylation sites are adjacent to the CXXC domain, which mediates DNA binding (Fig. 6c). Therefore, the potential influence of CFP1 phosphorylation on DNA-binding ability was evaluated by chromatin immunoprecipitation (ChIP) assay in HeLa cells transfected with plasmid expressing HA-CFP1 (WT, 2SD, and the non-DNA binding mutant C173A as a negative control). The binding of CFP1 with its reported target gene promoter regions^22,30^ was evaluated. For the four genes detected, CFP1^WT^ and CFP1^2SD^ had comparable binding ability with the genomic DNA, whereas the same genomic DNA fragments were not co-precipitated with CFP1^C173A^ (Supplementary Fig. 3e). These results confirmed that phosphorylation inhibited CFP1 by impairing its histone-binding, but not DNA-binding, ability.

### CFP1 inhibition are crucial for embryo development

**Fig. 10:**
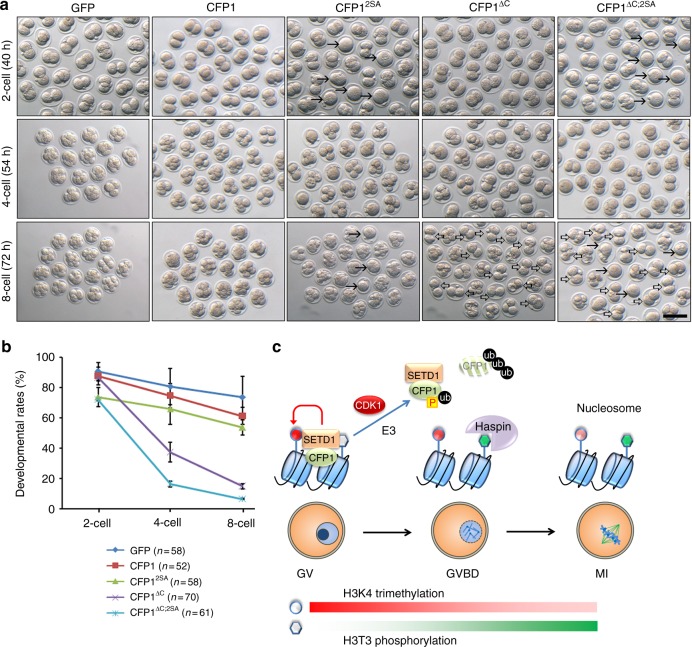
Inhibition of CFP1 phosphorylation and degradation blocks preimplantation embryo development. **a** In vitro development of wild-type mouse zygotes microinjected with mRNA encoding WT or mutated CFP1. Time after superovulation was indicated. Scale bar, 100 μm. Arrows and hollow arrows indicated embryos arrested at 1- and 2-cell stages, respectively. **b** Quantification of developmental rates in (**a**). Error bars, S.E.M. **c** A diagram showing the function and regulation of CFP1 during oocyte meiotic maturation. In GV oocytes, SETD1–CFP1 complex mediates accumulation of H3K4me3, which is a permissive signal for haspin-mediated H3T3 phosphorylation upon meiotic resumption. During meiotic resumption, CDK1 triggers CFP1 phosphorylation and degradation. Phosphorylation weakened the interaction between CFP1 and histone H3, and ubiquitin–proteasome pathway-mediated degradation removes CFP1 from the condensing chromosomes. This dual inhibition mechanism maintains an appropriate H3K4me3 level that is essential for meiotic cell cycle progression

We found that CFP1 is degraded not only in dividing mouse oocytes but also in blastomeres and somatic cells in metaphase of the cell cycle. To evaluate the physiological importance of CFP1 inhibition for mitosis, we overexpressed WT or mutated CFP1 in mouse zygotes by mRNA microinjection and monitored the preimplantation developmental process for the next 72 h. While overexpression of CFP1^WT^ in the zygote did not significantly affect embryo development to the 8-cell stage, the constitutively expressing CFP1 (CFP1^ΔC;2SA^) blocked embryo development beyond a 2-cell stage (Fig. 10a, b; arrows and hollow arrows indicated embryos arrested at 1- and 2-cell stages, respectively). In comparison, overexpression of CFP1^ΔC^ or CFP1^2SA^ caused intermediate phenotypes: although CFP1^ΔC^ decreased embryonic developmental rates to a lesser extent than CFP1^ΔC;2SA^ did, CFP1^2SA^ impaired the development from the 1- to 2-cell stage as significantly as CFP1^ΔC;2SA^ did but was less effective in the next two cell cycles (Fig. 10a, b). This is conceivable because the CFP1^2SA^ protein was degraded during the first mitotic division. Overall, these results indicated that dual inhibition of CFP1 by phosphorylation and degradation is not only important in oocytes but also crucial for successful preimplantation embryo development.

## Discussion

Although H3K4me3 is an evolutionarily conserved and common histone modification, its transcription-independent role in cell division has not been investigated before this study. Several research groups have demonstrated that the amount of H3K4me3 remarkably increases during oocyte maturation, and H3K4me3 is abundantly deposited at numerous broad regions on chromatin of mature oocytes^31–33^, but the functional significance of these phenomena remain unclear. It is well recognized that oocytes with an SN chromatin configuration have a better developmental potential than those with an NSN configuration^34^. This notion is in agreement with our finding that the H3K4me3 level in SN oocytes is higher than that in NSN oocytes. Therefore, H3K4me3 is a reliable marker of oocytes with high maturation rates and a post-fertilization developmental potential. Our results suggest that reinforcing H3K4me3 accumulation in human oocytes may be a promising approach to optimizing oocyte quality during human-assisted reproduction.

It is worth noticing that the adjacent threonine residue (T3) of H3K4 is modified by phosphorylation during cell division^35^. Mitotic and meiotic cell cycle–coupled phosphorylation of H3T3 by haspin kinase performs a pivotal function in spindle assembly and chromosome alignment^4,36,37^. The H3T3ph mark recruits survivin (a subunit of the chromosomal passenger complex) to chromatin and is essential for proper chromosome segregation and for modulation of aurora B kinase activity^38^. The haspin inhibitor 5-ITu was reported to impair spindle assembly and chromosome separation in mouse oocytes^3^. In our experiment, CFP1 deletion in mouse oocytes led to phenotypes similar to those in oocytes with inhibited haspin, including compromised GVBD and PB1 emission rates, defective spindle assembly, and failure of homologous chromosome separation. Evidently, meiotic maturation–coupled H3T3 phosphorylation was significantly decreased in *Cxxc1*^*oo−/−*^ oocytes. According to these observations, we hypothesized that CFP1-mediated H3K4 trimethylation is a priming signal for H3T3 phosphorylation and facilitates meiotic resumption (Fig. 10c). This previously unrecognized scheme may ensure proper spindle assembly and chromosome organization during cell division.

During further testing of our hypothesis, ectopic expression of a H3.3 mutant (H3.3^T3E^; which mimics H3T3 phosphorylation) partially rescued GVBD and spindle assembly, but not PB1 emission rates, in *Cxxc1*^*oo−/−*^ oocytes. The rescue effect was not very efficient possibly because it is difficult to control the expression level of H3.3^T3E^ in oocytes, and abnormally low and high H3T3ph levels are all detrimental to meiotic spindle assembly^4^. Besides, H3.3^T3E^ may not effectively mimic the precise chromosomal localization and function of H3T3ph.

Interplays between H3T3 phosphorylation and H3K4 methylation have been investigated before by biochemical studies, but contradictory results were obtained. Protein structural analyses reveals that the bulkiness of K4 methylation compromises H3 tail interactions with the narrow substrate-binding groove of haspin^5^. Correspondingly, in vitro kinase assay results indicated that H3K4 methylation impaired H3T3 phosphorylation by haspin^5^. This finding is consistent with the observation that H3T3ph was detected adjacent to, but not overlap with H3K4me3, on chromosome spreads of oocytes (Supplemental Fig. S3D of this study). In return, in vitro and in vivo results derived from nuclear magnetic resonance chemical shift perturbation analysis, fluorescence binding assays, pull-downs, peptide microarrays, and cell fluorescence microscopic studies revealed a strong anti-correlation between histone H3T3 phosphorylation and the ability of PHD finger-containing proteins (including CFP1) to recognize H3K4me3 or to retain in chromatin during mitosis^6^. Contradictorily, co-occurrence of H3T3ph/K4me, H3K9me/S10ph, and other phospho/methyl modifications has also been experimentally detected, and combinatorial PTM-specific antibody studies reveal that K4me3 and T3ph can be present on the same histone H3 tail^39,40^.

Our results revealed that H3K4 methylation affects H3T3 phosphorylation in an intermolecular manner because the ectopically expressed H3K4 mutant (H3.3^K4R^) decreased T3 phosphorylation of endogenous histone H3. Technically, we could not distinguish the intermolecular effects of monomethylation, dimethylation, and trimethylation of K4 on T3 phosphorylation in this study because all these modifications were abrogated in the H3.3^K4R^ mutant. Nonetheless, only trimethylation, but not the other modifications of H3K4, was significantly repressed in *Cxxc1*^*oo−/−*^ oocytes. Therefore, we concluded that H3K4me3, rather than H3K4me1 or H3K4me2, triggers H3T3 phosphorylation. In our previous study, a decreased H3K4me3 level in CFP1-null oocytes caused tightening of the chromatin structure and reduced the rate of histone H3 exchange^16^. As a result, the T3 sites in histone H3 on the chromatin of *Cxxc1*^*oo−/−*^ oocytes may not be accessible to haspin. Although our results indicate that defective T3 phosphorylation is an important reason for meiotic maturation failure of *Cxxc1*^*oo−/−*^ oocytes, it is not the only abnormality that matters here. Insufficient accumulation of maternal mRNAs and aberrant distribution of cytoplasmic organelles caused by CFP1 deletion may also indirectly influence meiotic cell cycle progression of oocytes^16^.

In this study, we observed that CFP1 is phosphorylated and degraded during the G2–M transition in both meiosis and mitosis. Chromatin undergoes extensive reorganization throughout the cell cycle, transitioning from a relatively relaxed state in interphase to a highly condensed state in mitosis and returning back to the interphase state after cell division is completed^27^. The degradation of CFP1 is a robust way to remove the SETD1–CFP1 complex from chromatin and therefore facilitates chromosome condensation. Otherwise, chromosome-bound CFP1 proteins, particularly those in the centromeric region, may become an obstacle for chromosome organization and microtubule attachment during spindle assembly. The finding of complete degradation of CFP1 during each cell division reveals a surprising fact that all SETD1–CFP1 H3K4 methyltransferase complexes need to be de novo assembled in the cell after each division, and the distribution pattern of this complex on chromatin needs to be re-established in every cell cycle. This knowledge is conceptually important for understanding the genome-wide regulation of H3K4 methylation dynamics in general. For instance, this concept implies that quickly proliferating cells tend to have a more dynamic H3K4me3 distribution pattern, whereas quiescent non-proliferating cells have relatively stable SETD1–CFP1 and H3K4me3 localization on chromatin.

Although our data indicate that CDK1 is involved in CFP1 degradation and phosphorylation, which ubiquitin E3 ligase directly binds to CFP1 and mediates its polyubiquitination is currently unclear. It is conceivable that this ubiquitin E3 ligase is activated by CDK1 during the G2–M transition and may directly interact with the C terminus of CFP1. Nor do we know the signal that recruits nondegradable CFP1 to the centromere region.

CDK1 is a multifunctional kinase and is essential for oocyte meiotic resumption^41,42^. Therefore, we could not test whether CDK1 inhibitors, such as roscovitine, prevent CFP1 phosphorylation and degradation, because the inhibitors would block oocyte GVBD as well. We cannot completely rule out the possibility that CDK1 activation leads to CFP1 degradation and phosphorylation indirectly as consequences of the G2–M transition. Nonetheless, our experiments showed that (1) CDK1 directly interacted with CFP1; (2) mutations at two putative CDK1 phosphorylation sites (S138 and S143) abrogated meiotic division–coupled CFP1 phosphorylation; and (3) inhibition of other G2–M transition–related kinases, such as haspin and aurora A^43^, did not prevent CFP1 phosphorylation. Together, these results strongly suggest that CDK1 directly phosphorylates CFP1.

Although CFP1 phosphorylation is not a prerequisite for CFP1 degradation, the dual inhibition of CFP1 is physiologically important. Stabilization of CFP1 in a maturing oocyte by C-terminal deletion did not completely prevent the reduction in H3K4me3 levels. Only when both CFP1 phosphorylation and degradation were abrogated, was H3K4me3 maintained at high levels in maturing oocytes, and strong defects in oocyte meiotic maturation were observed. The CFP1 phosphorylation sites are conserved among vertebrates and are close to the CxxC finger domain. Mutations that mimic phosphorylation (CFP1^2SD^) did not impair the SETD1–CFP1 interaction but weakened the chromatin binding of CFP1. In agreement with this observation, CFP1^2SD^ has a dominant-negative effect on H3K4 trimethylation in GV oocytes. Therefore, phosphorylation of CFP1 provides a mechanism for SETD1–CFP1 regulation and may be involved in fine-tuning of genomic histone modification processes. CFP1 is also degraded in mitosis, and constitutively active CFP1 blocks preimplantation murine embryo development. These facts suggest that the dual inhibition of the SETD1–CFP1 complex is a widespread mechanism in cell cycle regulation that is not restricted to oocytes. Furthermore, unidentified extracellular signals, ubiquitin E3 ligases, or kinases other than CDK1 may regulate activity of the SETD1–CFP1 complex via this dual inhibition mechanism in interphase cells in a time- and location-specific manner.

## Methods

### Animals

Three-to-four-week-old C57BL/6 mice maintained under specific pathogen-free conditions were used for all experiments. *Cxxc1*^*fl/fl*^;*Gdf9-Cre* mice were produced by crossing mice bearing the previously reported *Cxxc1*^*fl*^ allele with *Gdf9-Cre* transgenic mice^8,44^. Animal care and experimental procedures were in accordance with the Animal Research Committee guidelines of Zhejiang University.

### Oocyte culture

Female mice were primed with PMSG (5 IU) and full-grown oocytes were collected 44 h later by ovarian puncture. Healthy looking fully grown oocytes were collected from antral follicles and cultured in M16 medium (M7292; Sigma-Aldrich) covered with mineral oil at 37 °C in a 5% CO_2_ atmosphere^45^.

### Plasmid construction and IVT

cDNAs encoding human histone H 3.3 and CFP1 were subcloned into a Flag-tagged and a HA-tagged expression plasmid, respectively, and used for IVT. IVT was performed using the T7 and SP6 mMESSAGE mMACHINE Kit (Invitrogen, AM1344 and AM1340) according to the manufacturer’s instructions^46^.

### Immunofluorescence

Oocytes were fixed with 4% paraformaldehyde in phosphate-buffered saline (PBS) at room temperature for 30 min. They were then permeabilized with 0.3% Triton X-100 in PBS for 15 min. Antibody staining was performed using standard protocols described previously. Antibodies used in the experiments are described in Supplementary Table 1. Imaging was performed on a Zeiss LSM710 confocal microscope. Semiquantitative analysis of the fluorescence signals was conducted using the NIH Image program Image-J, as previously described^27^.

### Chromosome spreading

Zona pellucida-free oocytes were fixed in a solution containing 1% paraformaldehyde, 0.15% Triton X-100, and 3 mmol/L dithiothreitol (Sigma-Aldrich) on glass slides for 30 min and air dried. Immunofluorescent staining was performed as in oocytes described above.

### Microinjection of mRNAs and small interfering RNAs

All microinjections were performed using an Eppendorf transferman NK2 micromanipulator. Fully grown GV oocytes were harvested in M2 medium with 2 μM milrinone to inhibit spontaneous GVBD. Approximately 5 pl of 200 μg/ml mRNAs was microinjected into the ooplasm.

### Live cell imaging

For live imaging, mRNAs encoding for GFP-tubulin and mCherry-securin were microinjected into WT and CFP1-deleted oocytes and released from milrinone after 8 h. Oocytes were cultured in M16 medium containing Hoechst-33342 (to label DNA) and subjected to live cell imaging at 5 h after meiotic resumption. Live oocytes were acquired on a DV ELITE High Resolution Invented Living Cell Work station. Image acquisition was performed using Zeiss LSM-780 confocal microscope (Zeiss) equipped with PC-Apochromat 20×/0.8 NA objective lenses at 6 min intervals for 13 h.

### Immunoblot

Oocytes were lysed in protein-loading buffer and heated at 95 °C for 5 min. SDS-PAGE and immunoblots were performed following the standard procedures using a Mini-PROTEAN Tetra Cell System (Bio-Rad, Hercules, CA). The antibodies used are listed in Supplementary Table 1. Uncropped scans of the most important blots are supplied as Supplementary Figures 4, 5.

### Cell culture

HeLa cells were from the American Type Culture Collection and were grown in Dulbecco’s modified Eagle’s medium (Invitrogen) supplemented with 10% fetal bovine serum (Hyclone) and 1% penicillin–streptomycin solution (Gibco) at 37 °C in a humidified 5% CO_2_ incubator. Cells were in healthy condition but were not tested for mycoplasma contamination.

### Immunoprecipitation assay

The immunoprecipitation (ChIP) assay in HeLa cells was performed using the SimpleChIP Enzymatic Chromatin IP Kit (CST, 9003) according to the manufacturer’s protocol. Approximately 4 × 10^6^ cells were used for each immunoprecipitation. Immunoprecipitation of immunoglobulin G (CST#2729; 1 µg, as a negative control) or HA (CST#3724; 1 µg per sample) was performed at 48 h after transfection. In all, 10% total DNA was used for input evaluation. PCR products of immunoprecipitated and input samples were analyzed on a 2% agarose gel. Sequences of primers for PCR amplification are provided in Supplementary Table 2.

### Statistical analysis

Two-tailed Student’s *t* test was used to calculate *P* values. Statistically significant values for <0.05, <0.01, and <0.001 are indicated by single, double, and triple asterisk, respectively.

### Data availability

The authors declare that all data supporting the findings of this study are available within the article and its supplementary information files or from the corresponding author upon reasonable request.

## Electronic supplementary material

Supplemental information

Peer Review File
