# Supplementary material for: CFP1 coordinates histone H3 lysine-4 trimethylation and meiotic cell cycle progression in mouse oocytes
Source: Nat Commun. 2018 Aug 28;9:3477. doi: 10.1038/s41467-018-05930-x (PMC6113306; doi:10.1038/s41467-018-05930-x)
Supplement: Supplementary file 1 — Supplemental information [file 41467_2018_5930_MOESM1_ESM.pdf]

Supplementary Information

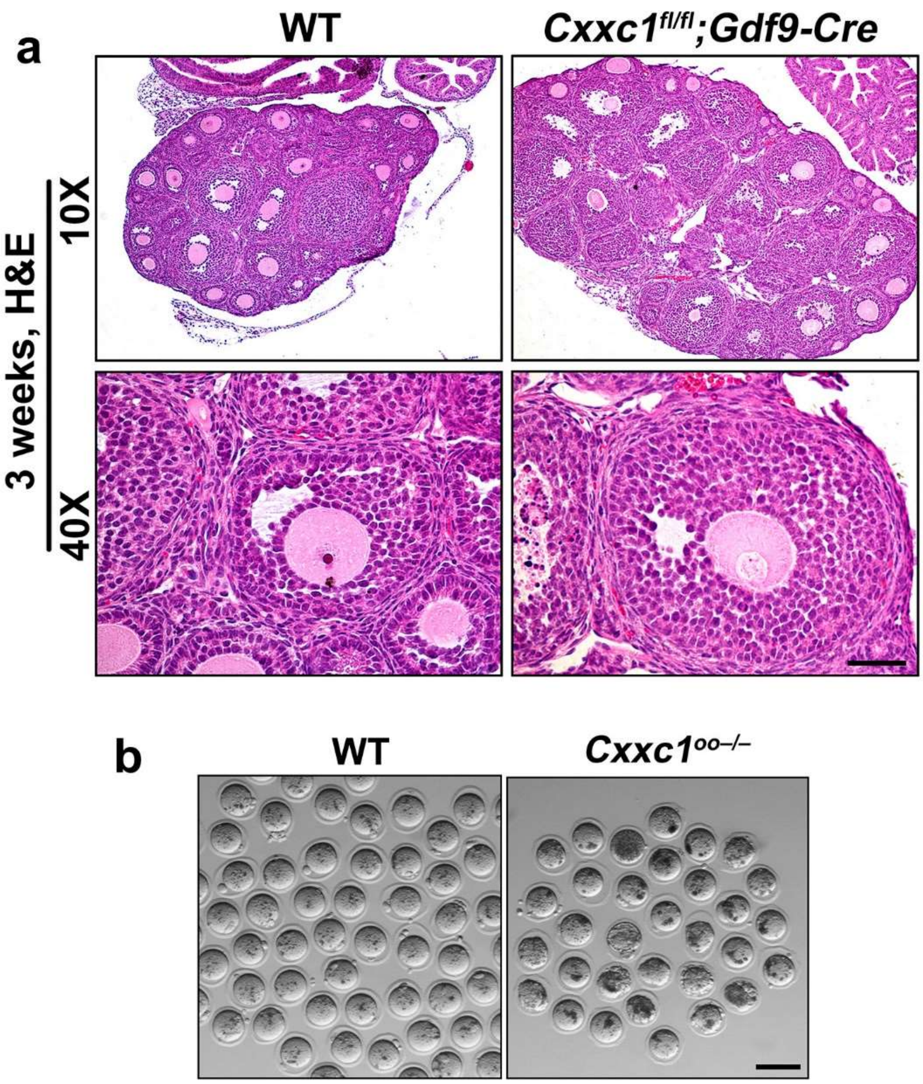

Supplementary Figure 1. Phenotype analyses of *Cxxc1$^{fl/fl}$;Gdf9-Cre* female mice.

a: H&E staining results showing ovarian histology of 3-week-old WT and *Cxxc1$^{oo-/-}$* mice. Scale bar, 50 μm. **b:** Representative images of oocytes ovulated by 3-week-old WT and *Cxxc1$^{oo-/-}$* mice, at 16 h after hCG injection. Scale bar, 100 μm.

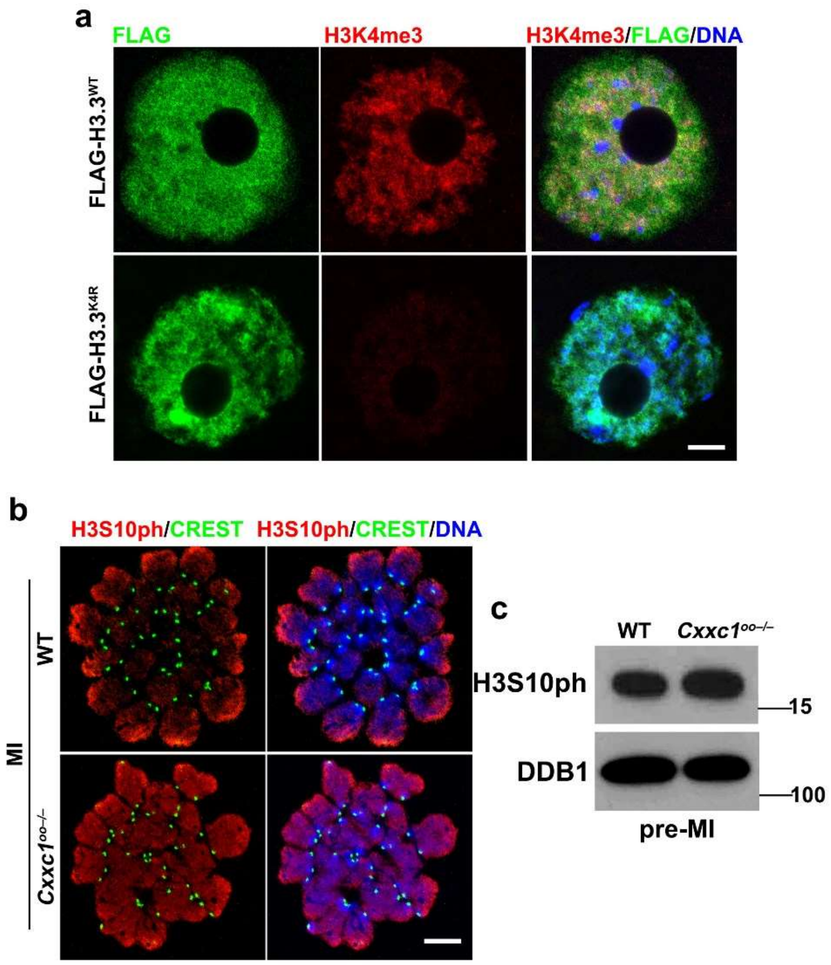

Supplementary Figure 2. Changes of histone H3 modifications after inhibition of H3K4 methylation.

a: Immunofluorescence results showing H3K4me3 levels in GV oocytes overexpressing Flag-tagged histone H3.3 and its K4R mutant, at 12 h after mRNA microinjection. Scale bar, 5 µm. **b-c:** Immunofluorescence (**b**) and Western blot (**c**) results showing levels of phosphorylated histone H3 at Ser-10 (H3S10ph) in WT and *Cxxc1*$^{oo-/-}$ oocytes at metaphase I. Scale bar, 5 µm.

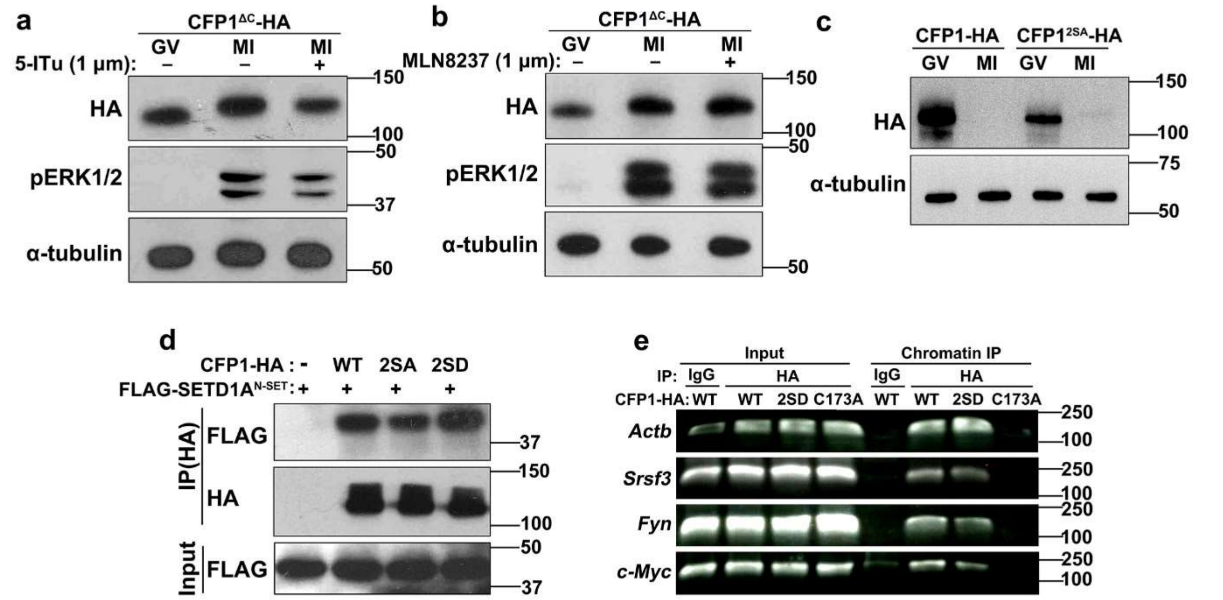

Supplementary Figure 3. Phosphorylation of CFP1 and its potential biochemical functions.

a-b: Western blot results showing CFP1 band shift in maturing oocytes. GV oocytes were microinjected with mRNA encoding CFP1$^{\Delta C}$-HA and cultured in medium containing milrinone for 3 h, and then were transferred to milrinone-free medium containing 5-ITu (**a**) or MLN8273 (**b**) for further culture (6 h). Total proteins from 100 oocytes were loaded in each lane. **c:** CFP1 degradation is independent of its phosphorylation in maturing oocytes. GV oocytes were microinjected with mRNA encoding HA-tagged CFP1^{WT} or CFP1^{2SA}, cultured in medium containing milrinone for 3 h, and then were transferred to milrinone-free medium for further culture (6 h). **d:** Co-immunoprecipitation results showing that phosphorylation of CFP1 does not affect its binding with SETD1. HeLa cells were co-transfected with plasmids expressing CFP1 (WT and phosphorylation site mutants) and the N-SET domain of human SETD1A. Cells were lysed at 48 h after transfection and used for immunoprecipitation and Western blots. **e:** Immunoprecipitation (ChIP) assay in HeLa cells transfected with plasmid expressing CFP1-HA (WT, 2SD, and C173A). ChIP of IgG (1 μg, as a negative control) or HA (1 μg per sample) was performed at 48 h after transfection. 10% DNA was used for input evaluation. The levels of indicated DNA fragments in input samples and immunoprecipitants were analyzed by PCR using primers described in Supplemental Table-2.

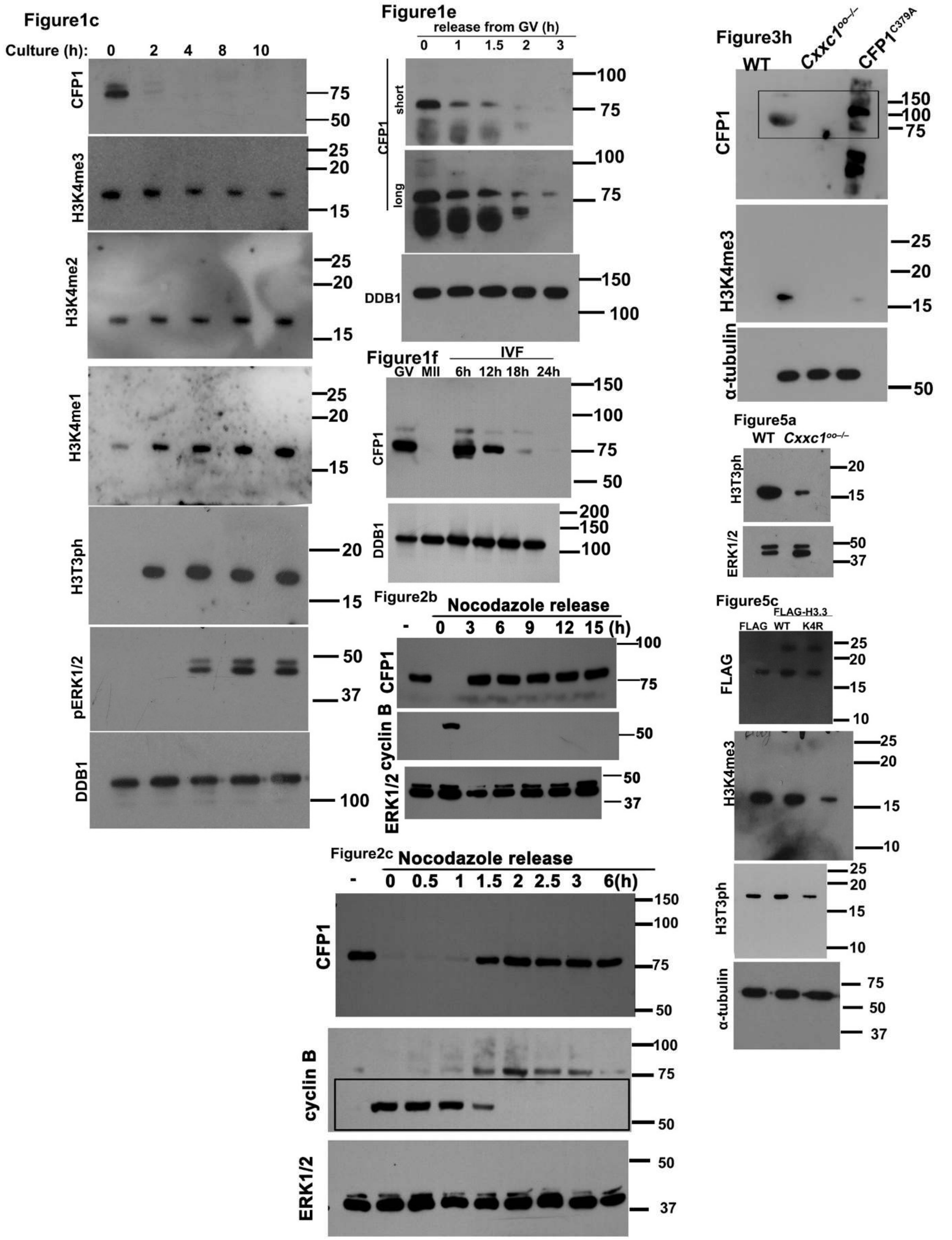

Supplementary Figure 4. Uncropped scans of the most important Western blot results in Figure 1-5. Black boxes highlight lanes used in figures.

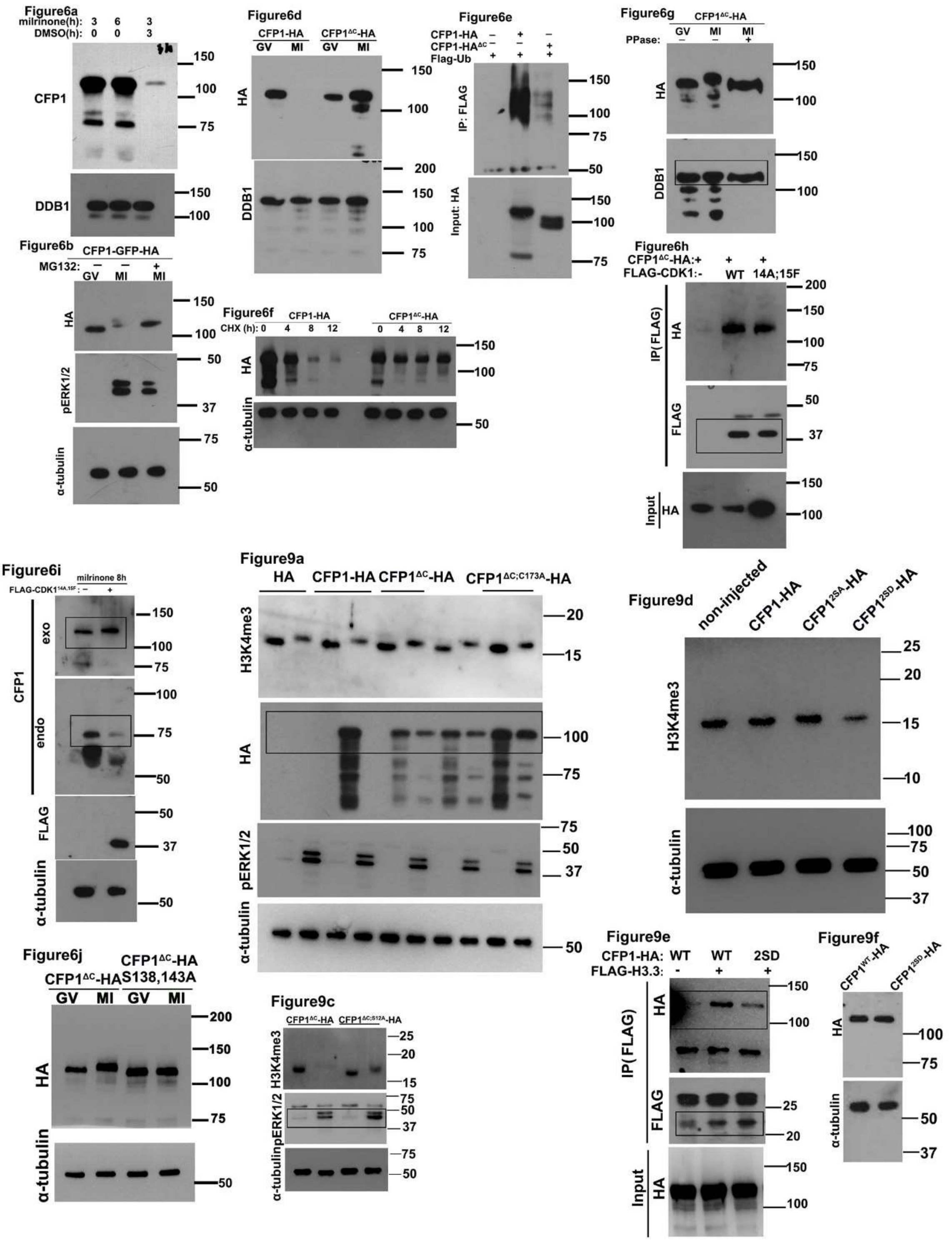

Supplementary Figure 5. Uncropped scans of the most important Western blot results in Figure 6 and 9. Black boxes highlight lanes used in figures.

Supplementary Table 1. Antibody information

Protein name	Manufacture (catalogue number)	Applications (working dilution)	Website Link*
**H3K4me1**	Cell Signaling (5326)	WB (1:1000)	http://www.cst-c.com.cn/products/primary-antibodies/mono-methyl-histone-h3-lys4-d1a9-xp-rabbit-mab/5326?_=1508331672321&Ntt=&tahead=true
**H3K4me2**	Cell Signaling (9725)	WB (1:1000)	http://www.cst-c.com.cn/products/primary-antibodies/di-methyl-histone-h3-lys4-c64g9-rabbit-mab/9725?_=1508331697704&Ntt=H3K4me&tahead=true
**H3K4me3**	Abcam (ab8580)	IF (1:400) WB (1:1000)	http://www.abcam.com/histone-h3-tri-methyl-k4-antibody-chip-grade-ab8580.html
**H3K4me3**	Gene Tex (RM137)	IF (1:400)	http://www.genetex.com/Histone-H3K4me3-Trimethyl-Lys4-antibody-RM137-GTX60876.html
**FITC-α-Tubulin**	Sigma (F2168)	IF (1:500)	http://www.sigmaaldrich.com/catalog/product/sigma/f2168?lang=zh&region=CN
**TOP2B**	Abcam (ab109524)	IF (1:200)	http://www.abcam.com/Topoisomerase-II-beta-antibody-EPR5377-ab109524.html
**CREST**	Fitzgerald Industries International (70R-21494)	IF (1:100)	https://www.fitzgerald-fii.com/crest-antibody-70r-21494.html
**SMC3**	Abcam (ab128919)	IF (1:20)	http://www.abcam.cn/smc3-antibody-epr7984-ab128919.html
**HA**	Cell Signaling (3724)	WB (1:1000) IF (1:400)	http://www.cst-c.com.cn/products/3724.html
**FLAG**	Sigma (F3165)	WB (1:3000) IF (1:400)	http://www.sigmaaldrich.com/catalog/product/sigma/f3165?lang=zh&region=CN
**ERK1/2**	Santa Cruz (sc-94)	WB (1:1000)	http://www.scbt.com/datasheet-94-erk-1-k-23-antibody.html
**p-ERK1/2**	Cell Signaling (9101)	WB (1:1000)	http://www.cellsignal.com/products/primary-antibodies/phospho-p44-42-mapk-erk1-2-thr202-tyr204-antibody/9101
**DDB1**	Epitomics (3821-1)	WB (1:10000)	http://www.epitomics.com/products/product_info/2283
**CFP1**	Abcam (ab198977)	WB (1:500); IF (1:100)	http://www.abcam.com/cgbp-antibody-epr19199-ab198977.html
**Cyclin B**	Cell Signaling (4138)	WB (1:1000)	http://www.cst-c.com.cn/products/primary-antibodies/cyclin-b1-antibody/4138?_=1510562752707&Ntt=&tahead=true

**p H3T3**	Abcam (ab78351)	IF (1:400)	http://www.abcam.cn/histone-h3-phospho-t3-antibody-ep1702y-ab78351.html
		WB(1:1000)	
**p H3S10**	Cell Signaling (9701S)	IF (1:100)	http://www.cst-c.com.cn/products/9701.html
		WB (1:1000)	

Supplementary Table2: Sequences of primers (5'-3') used to amplify genomic DNA fragments in Chromatin immunoprecipitation assay.

**Primer name**	**Genes targeted**	**Sequences (5'-3')**
Actb -F	*Actb*	CCAGGCTGGCTTTGAGTTCC
Actb -R		GCCCACCGTCCGTTGTATG
Srsf3-F	*Srsf3*	TTTGGCTTATGTAGGTACTTTG
Srsf3-R		GGAGCATTTACCACCATCTG
Fyn -F	*Fyn*	TCCTCCTCATTGTTATTTGTTGTG
Fyn R		GGGCTATCTTGCCTTTCTGG
c-Myc -F	*c-Myc*	TTTCGGCTCACCGCATTTC
c-Myc -R		CCTACCCAACACCACGTCCTA
